# *Stevia Eupatoria* and *Stevia Pilosa* Extracts Inhibit the Proliferation and Migration of Prostate Cancer Cells

**DOI:** 10.3390/medicina56020090

**Published:** 2020-02-23

**Authors:** Elizabeth Martínez-Rojo, Raquel Cariño-Cortés, Laura Cristina Berumen, Guadalupe García-Alcocer, Jesica Escobar-Cabrera

**Affiliations:** 1 Unidad de Investigación Genética, Posgrado en Ciencias Químico Biológicas, Facultad de Química, Universidad Autónoma de Querétaro, Centro Universitario, Querétaro 76010, Mexico; elizaro4309@gmail.com (E.M.-R.); lcbsq@yahoo.com (L.C.B.); guadalugar@yahoo.com.mx (G.G.-A.); 2 Centro de Investigación en Biología de la Reproducción del Instituto de Ciencias de la Salud, Universidad Autónoma del Estado de Hidalgo, Pachuca 42082, Hidalgo, Mexico; raquelcarcortes@gmail.com

**Keywords:** *Stevia pilosa*, *Stevia eupatoria*, enzalutamide, prostate cancer, proliferation, migration

## Abstract

*Background and Objectives*: Prostate cancer is the second most harmful disease in men worldwide and the number of cases is increasing. Therefore, new natural agents with anticancer potential should be examined and the response of existing therapeutic drugs must be enhanced. *Stevia pilosa* and *Stevia eupatoria* are two species that have been widely used in traditional medicine, but their effectiveness on cancer cells and their interaction with antineoplastic drugs have not been studied. The aim of this study was to evaluate the anticancer activity of *Stevia pilosa* methanolic root extract (*SPME*) and *Stevia eupatoria* methanolic root extract *(SEME)* and their effect, combined with enzalutamide, on prostate cancer cells. *Materials and Methods:* The study was conducted on a human fibroblast cell line, and on androgen-dependent (LNCaP) and androgen-independent (PC-3) prostate cancer cell lines. The cell viability was evaluated using a Trypan Blue exclusion test for 48 h, and the migration by a wound-healing assay for 24, 48, and 72 h. *Results:* The results indicate that *SPME* and *SEME* were not cytotoxic at concentrations less than 1000 μg/mL in the human fibroblasts. *SPME* and *SEME* significantly reduced the viability and migration of prostate cancer cells in all concentrations evaluated. The antiproliferative effect of the *Stevia* extracts was higher in cancer cells than in normal cells. The enzalutamide decreased the cell viability in all concentrations tested (10–50 µM). The combination of the *Stevia* extracts and enzalutamide produced a greater effect on the inhibition of the proliferation and migration of cancer cells than the *Stevia* extracts alone, but not of the enzalutamide alone. *Conclusion:* The results indicate that *SPME* and *SEME* have an inhibitory effect on the viability and migration of prostate cancer cells and do not interfere with the enzalutamide anticancer effect. The data suggest that *Stevia* extracts may be a potential source of molecules for cancer treatment.

## 1. Introduction

Prostate cancer (PCa) is one of the most frequent causes of death in men aged over 65 years old around the world [1,2]. PCa development and progression depend on the androgen receptor pathway; the androgen receptor (AR) is a member of the steroid and nuclear hormone receptor superfamily. The main circulating androgen is testosterone, produced in the testes and converted to dihydrotestosterone (DHT) by 5α-reductase in the prostate. The AR pathway begins when DHT binds to AR and induces a conformational change resulting in the dissociation of cytoplasmic chaperones. The hormone-bound AR dimerizes and translocates to the nucleus where it binds to DNA and interacts with transcriptional co-regulators to moderate the expression of genes involved in proliferation and survival [3]. Based on the crucial role played by androgens and AR, hormonal therapy is one type of treatment available for prostate cancer [3,4], and the second-generation antiandrogen enzalutamide has been approved for castration-resistant prostate cancer. Enzalutamide inhibits the AR pathway in three steps: by inhibiting receptor binding by DHT, by blocking DHT–AR nuclear translocation, and by interfering with the AR-mediated transcription of genes involved in proliferation and survival. In this way, enzalutamide decreases the proliferation of cancer cells [3,5,6,7]. Some PCa patients develop castration-resistant prostate cancer and later develop metastases. Few treatments are approved, like enzalutamide, that delay the progression of the illness while maintaining a good quality of life [8]. However, the resistance to therapy and risk of recurrence has led to the search for new effective anticancer compounds, including those from medicinal plants [9]. *Stevia* is a perennial herbaceous shrub that grows from the Southwest USA to Northern Argentina and is traditionally used as a medicinal plant [10]. Of all the *Stevia* species that exist, approximately 70 species are native to Mexico, and their anticancer activity has rarely been studied. *Stevia pilosa* and *Stevia eupatoria* are used for the treatment of stomach pains as a hypoglycemic agent, and as a diuretic, analgesic, anti-inflammatory, antihypertensive, and mutagenesis protector in cells exposed to alkylating agents [11]. These effects are attributed to their compounds such as flavonoids, sterols, sesquiterpenes, and longipinenes, which possess important biological activities [12]. Some species of *Stevia* have demonstrated their effect on the antiproliferative activity in cervical, pancreatic, colon, breast, and glioma cancer cells [10,13,14]. However, the effects of *Stevia pilosa* and *Stevia eupatoria* on the proliferation and migration of cancer cells has not yet been evaluated. Therefore, the aim of this study was to evaluate the effects of *Stevia pilosa* methanolic root extract (*SPME*) and *Stevia eupatoria* methanolic root extract (*SEME*) alone and in combination with enzalutamide on the cell viability and migration of androgen-dependent (LNCaP) and androgen-independent (PC-3) cell lines.

## 2. Materials and Methods

### 2.1. Materials

The human fibroblasts (HDFn) and LNCaP cell lines were provided by Dr. Claudia Lucia Vargas Requena (Biotechnology Lab, Universidad Autónoma de Ciudad Juárez, Juárez, Chihuahua, México) and Dr. Brenda Anguiano (Instituto de Neurobiología, Universidad Nacional Autónoma de México, Querétaro, Querétaro, México), respectively. The PC-3 cell line was obtained from ATCC (Manassas, VA, USA). The *SPME* and *SEME* were provided by Dr. Raquel Cariño Cortés (Toxicology Laboratory, Universidad Autónoma del Estado de Hidalgo, Pachuca de Soto, Hidalgo, México). Dulbecco’s modified Eagle’s medium (DMEM), Kaighn´s modification of Ham´s F-12 Medium (F-12K), Roswell Park Memorial Institute (RPMI) 1640 medium, and trypsin were obtained from Corning (Manassas, VA, USA). The enzalutamide was purchased from MedChem Express (Monmouth Junction, NJ, USA). The methodology of this project was approved on 15 December, 2017 by the bioethics committee of Facultad de Química, Universidad Autónoma de Querétaro with the ethical code number CBQ17/101.

### 2.2. Cell Culture and Cell Proliferation Assay

The HDFn, LNCaP, and PC-3 cells were maintained in DMEM, RMPI 1640, and F-12K media, respectively, supplemented with 10% fetal bovine serum (FBS) and 100 IU/mL penicillin in a humidified incubator containing 5% CO_2_ at 37 °C. For the cell proliferation assay, 5 × 10^4^ cells/well were seeded in a 24-well plate and incubated at 37 °C and 5% CO_2_ for 48 h. After this, the cells were washed with phosphate-buffered saline (PBS) and incubated with different concentrations of *SPME* or *SEME* (0, 250, 500, 1000, 2000, 2500 and 3000 μg/mL) for 48 h. The enzalutamide was evaluated at different concentrations (0, 10, 20, 30, 40 and 50 μM) for 48 h. For combinatorial treatments, the cells were incubated with 40 μM enzalutamide + 500 μg/mL of extract (*SPME* or *SEME*) or 40 μM enzalutamide + 1000 μg/mL of extract (*SPME* or *SEME*) for 48 h. After the treatment, the cells were washed with PBS, trypsinized, and counted with Trypan Blue 0.04% in a Neubauer chamber. All of the experiments were performed in triplicate, at least.

### 2.3. Wound Healing Assay

The wound-healing assay was conducted to measure the migratory capacity of PCa cells. The PC-3 cells (2 × 10^5^) were seeded in a 6-well plate and grew until they formed a confluent monolayer. The monolayers were scratched with three wounds per well and washed with PBS. Then, for the individual treatments, the cells were treated with different concentrations of *SPME* or *SEME* (250, 1000, and 2500 μg/mL). For the combinatorial treatment, the cells were incubated with 40 μM enzalutamide + 1000 μg/mL of *SPME* or *SEME*. The wound closure was evaluated at 0, 24, 48, and 72 h after exposure to extracts. The migration rate was calculated as follows: Migration rate = (Average distance between wound (at 0 h)—average distance between wound (at 72 h))/Average distance between wound (at 0 h). We calculated the wound width by measuring the distance between 3 random points within the wound edges.

### 2.4. Statistical Analysis

Data were analyzed with Graph Pad Software Prism 6.0 (San Diego, CA, USA) and expressed as mean + standard error of the mean (S.E.M.) of three independent experiments. Data were compared among groups using a one-way analysis of variance (ANOVA) with a Tukey test used for post-hoc comparisons. The statistical significance was set at *P* < 0.05.

## 3. Results

### 3.1. Effect of Stevia Pilosa and Stevia Eupatoria on the Proliferation of Human Fibroblasts

**Figure 1:**
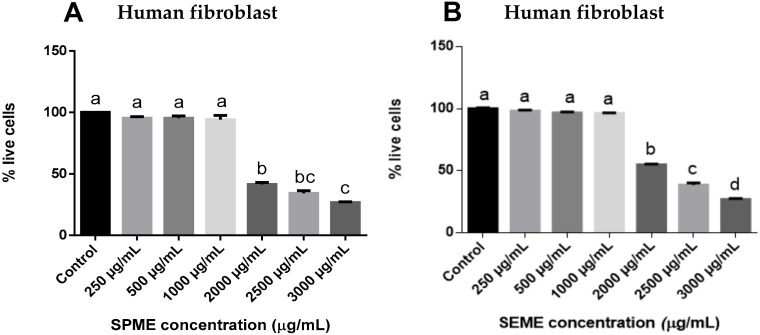
Effect of *Stevia* extracts on the proliferation of human fibroblasts. Human fibroblasts treated with (**A**) *Stevia pilosa* methanolic root extrac (*SPME*) and (**B**) *Stevia eupatoria* methanolic root extrac (*SEME*). Cells were treated for 48 h. Values are expressed as mean ± standard error of the mean (S.E.M.) Values with different letters (a–d) are significantly different (*P* < 0.05). Experiments were performed three times.

To determine whether *SPME* and *SEME* have a cytotoxic effect on untransformed cells, the human fibroblasts were treated with both extracts. As a result, the proliferation of treated cells with 250, 500 and 1000 μg/mL did not show a significant difference with respect to the control. The cytotoxicity was observed from the concentration of 2000 μg/mL to decrease the percentage of live cells by 58.6% with 2000 μg/mL, 65.7% with 2500 μg/mL, and 73.3% with 3000 μg/mL of *SPME* (Figure 1A); and 45.2% with 2000 μg/mL, 61.5% with 2500 μg/mL, and 73.1% with 3000 μg/mL of *SEME* (Figure 1B).

### 3.2. Stevia Pilosa and Stevia Eupatoria Inhibited Proliferation of Prostate Cancer Cell Lines

**Figure 2:**
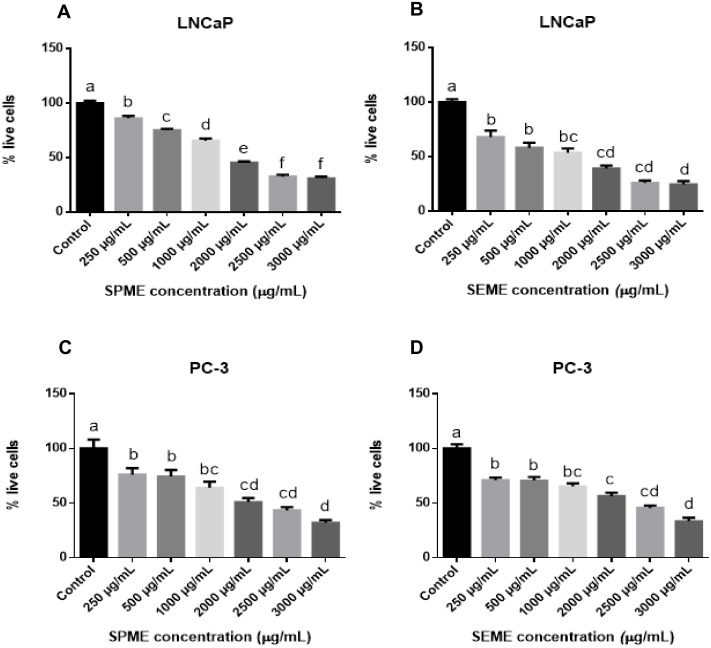
Effect of *SPME* and *SEME* on proliferation in prostate cancer cell lines. LNCaP cells treated with (**A**) *SPME* and (**B**) *SEME*; PC-3 cells treated with (**C**) *SPME* and (**D**) *SEME*. Cells were treated for 48 h with the extracts. Values are expressed as mean ± S.E.M. Values with different letters (a–e) are significantly different (*P* < 0.05). Experiments were performed three times.

We treated androgen-dependent cells (LNCaP) and androgen-independent cells (PC-3) with different concentrations of both extracts for 48 h. The concentrations of 250, 500, 1000, 2000, 2500, and 3000 μg/mL of *SPME* or *SEME* inhibited proliferation in a concentration-dependent manner. *SPME* produced inhibition rates of 13.1%, 26.1%, 34.8%, 52.2%, 65.3%, and 69.6% in LNCaP cells (Figure 2A) and 30.4%, 30.4%, 34.7%, 43.5%, 56.5%, and 69.6% in PC-3 cells, respectively (Figure 2C). *SEME* also inhibited proliferation, showing inhibition rates of 17.4%, 26%, 34.7%, 47.8%, 56.5%, and 69.5% in LNCaP cells (Figure 2B), and 30.4%, 43.5%, 47.8%, 60.9%, 73.9%, and 78.3% in PC-3 cells, respectively (Figure 2D).

### 3.3. Effect of the Combinatorial Treatment of Stevia Extracts with Enzalutamide on LNCaP and PC-3 Cell Viability

**Figure 3:**
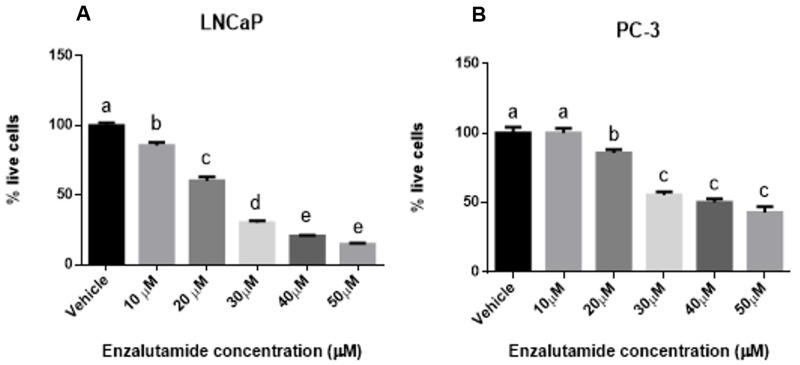
Enzalutamide effect on viability of prostate cancer cells. (**A**) LNCaP cells and (**B**) PC-3 cells exposed to enzalutamide for 48 h. Values are expressed as mean ± S.E.M. Values with different letters (a–e) are significantly different (*P* < 0.05). Experiments were performed three times.

**Figure 4:**
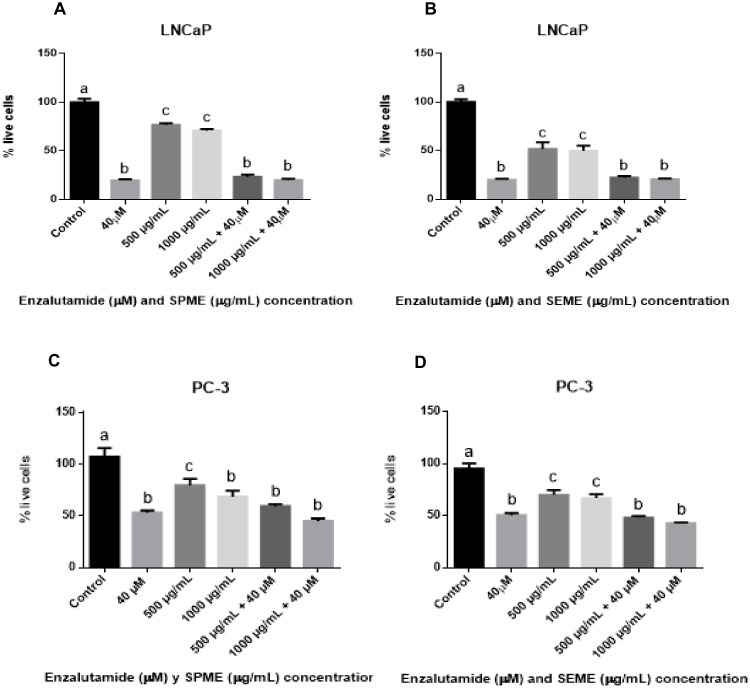
Effect of combinatorial treatment on the proliferation of prostate cancer cells. (**A**) LNCaP cells treated with enzalutamide and *SPME*, (**B**) LNCaP cells treated with enzalutamide and *SEME*, (**C**) PC-3 cells treated with enzalutamide and *SPME*, and (**D**) PC-3 cells treated with enzalutamide and *SEME*. Cells were treated with 40 μM enzalutamide + 500 μg/mL *SPME* or *SEME*, and 40 μM of enzalutamide + 1000 μg/mL of *SPME* or *SEME* for 48 h. Values are expressed as mean ± S.E.M. Values with different letters (a–c) are significantly different (*P* < 0.05). Experiments were performed three times.

We first evaluated the inhibitory effect of enzalutamide in the LNCaP and PC-3 cells, which showed a dose-dependent decrease in viability. Figure 3A shows a decrease at 10 μM of 14.4%, 20 μM of 39.8%, 30 μM of 69.8%, 40 μM of 79.6%, and 50 μM of 85% for LNCaP cells, whereas Figure 3B indicates a reduction at 20 μM of 14.2%, 30 μM of 44.3%, 40 μM of 49.6%, and 50 μM of 57.1% for PC-3 cells. The LNCaP cells showed lower viability than the PC-3 cells treated with enzalutamide. For combinatorial treatment, the cells were incubated with 40 μM of enzalutamide + 500 μg/mL of *SPME* or *SEME,* and 40 μM of enzalutamide + 1000 μg/mL of *SPME* or *SEME*. The cells treated with the combination of *Stevia* extracts and enzalutamide and the cells treated with enzalutamide alone showed the same viability; enzalutamide treatment alone reduced viability by 80%, 40 μM enzalutamide + 500 μg/mL *SPME* reduced viability by 76%, and 40 μM enzalutamide + 1000 μg/mL *SPME* reduced viability by 80% of LNCaP cells (Figure 4A). The cell viability with 40 μM enzalutamide + 500 μg/mL *SEME* was reduced by 77%; for cells treated with 40 μM enzalutamide + 1000 μg/mL *SEME*, viability was reduced by 79% of LNCaP cells (Figure 4B). A similar effect was observed in the PC-3 cells: enzalutamide reduced viability until 47.5%, whereas combinatorial treatments showed a reduction of 40.1% with 40 μM enzalutamide + 500 μg/mL *SPME* and 54.8% with 40 μM enzalutamide + 1000 μg/mL *SPME* (Figure 4C). The cell viability was reduced by 51.6% with 40 μM enzalutamide + 500 μg/mL *SEME* and by 57.2% with 40 μM enzalutamide + 1000 μg/mL *SEME* (Figure 4D). We found no significant difference between enzalutamide treatment alone and the combinations. The LNCaP cells showed a higher sensitivity to the treatments than the PC-3 cells.

### 3.4. Stevia Pilosa and Stevia Eupatoria Inhibited PC-3 Cell Migration

**Figure 5:**
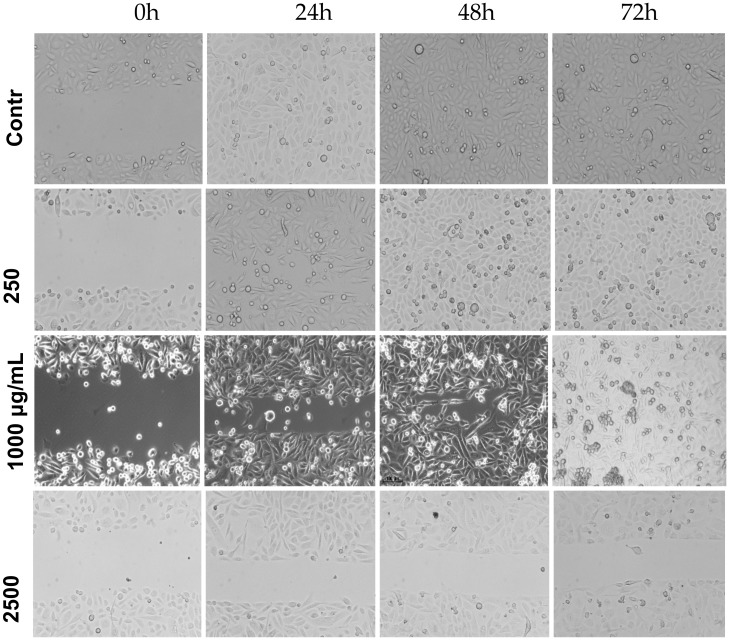
Effect of the *Stevia pilosa* on PC-3 cell migration. Cells were treated with *SPME* in different concentrations for 24, 48 and 72 h. Experiments were performed at least three times.

**Figure 6:**
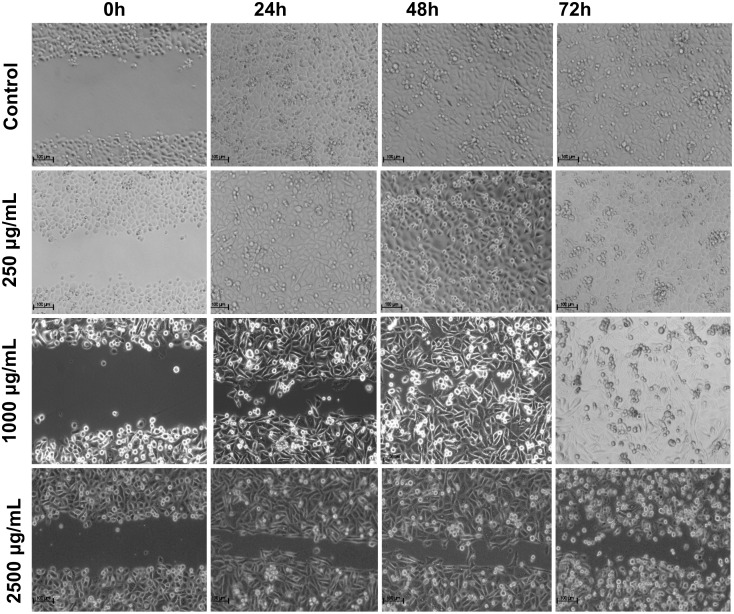
Effect of the *Stevia eupatoria* on PC-3 cell migration. Cells were treated with *SEME* in different concentrations for 24, 48, and 72 h. Experiments were performed at least three times.

**Figure 7:**
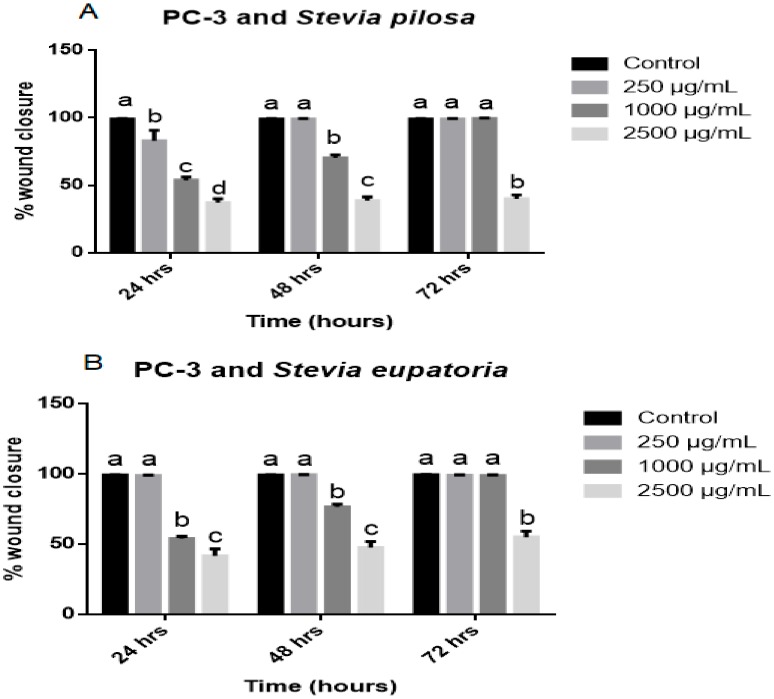
Wound closure rate of PC-3 cells’ migration exposed to *Stevia* extracts. (**A**) *SPME* and (**B**) *SEME* effects on PC-3 migration. Values are expressed as mean ± S.E.M. Values with different letters (a–e) are significantly different (*P* < 0.05). Experiments were performed at least three times.

We used a wound-healing assay to evaluate the effect of *SPME* and *SEME* extracts alone on the PC-3 cells. In control cells, the wound closed at 24 h for both extracts, indicating cell migration under normal conditions; cells treated with 250 μg/mL *SPME* closed the wound at 48 h; with 1000 μg/mL, the wound closed at 72 h; and with 2500 μg/mL, the wound did not close at any time evaluated (Figure 5). The results for *SEME* showed that wound closure for cells treated with 250 μg/mL occurred at 24 h, cells with 1000 μg/mL treatment showed wound closure at 48 h, and the 2500 μg/mL treatment prevented wound closure at all times evaluated (Figure 6). The migration rate of cells treated with *SPME* and *SEME* was calculated. In all concentrations tested, *SPME* significantly inhibited the migration of the PC-3 cells at 24 h with a concentration-dependent response. At 48 h of exposure, only 1000 and 2500 μg/mL displayed an inhibition of migration, and at 72 h, only 2500 μg/mL had an anti-migratory effect. The higher concentration of *SPME* (2500 μg/mL) inhibited the migration at all times tested, with values of 37% at 24 h, 38% at 48 h, and 40% at 72 h (Figure 7A). *SEME* significantly inhibited the PC-3 cell migration with 1000 and 2500 μg/mL at 24 and 48 h of exposure compared to the control, and at 72 h, only 2500 μg/mL had an anti-migratory effect. The higher concentration of *SEME* (2500 μg/mL) inhibited the migration at all times tested, with values of 42% at 24 h, 48% at 48 h, and 54% at 72 h (Figure 7B).

### 3.5. Combinatorial Effect of Stevia Extracts with Enzalutamide on PC-3 Cell Migration

**Figure 8:**
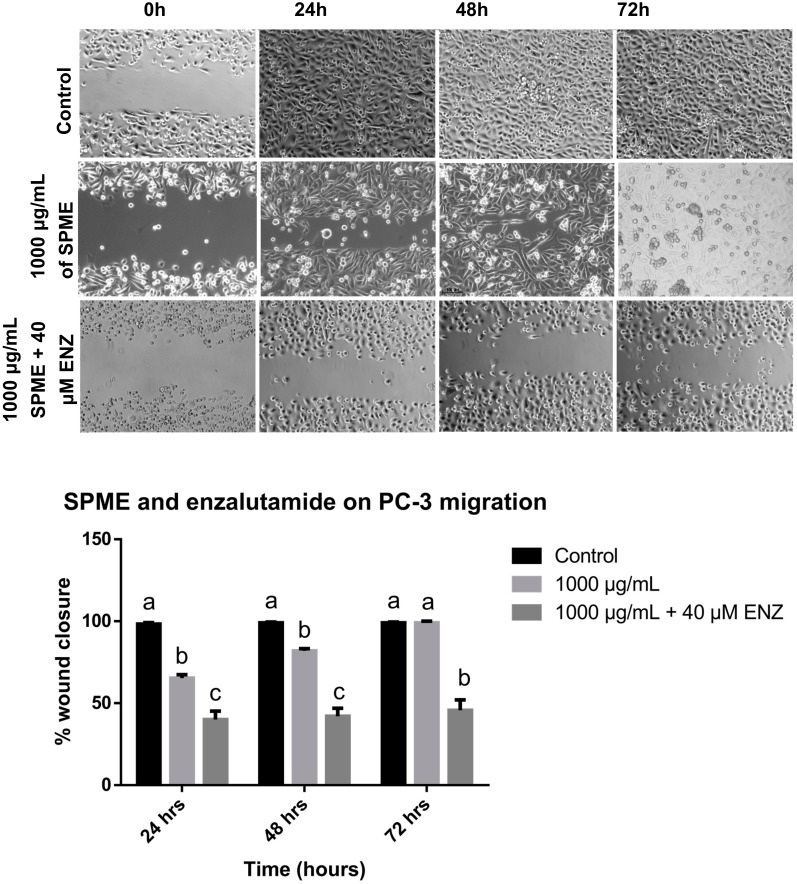
Effect of *SPME* in combination with enzalutamide on PC-3 cell migration. Micrography and statistical analysis of the wound healing assay. Values are expressed as mean ± S.E.M. Values with different letters (a–c) are significantly different (*P* < 0.05). Experiments were performed three times.

**Figure 9:**
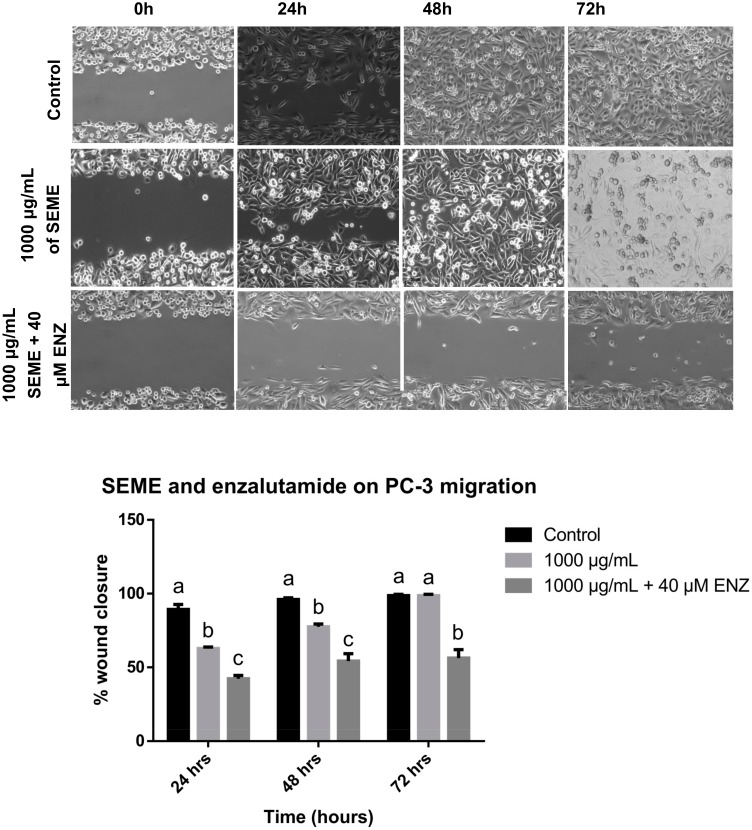
Effect of *SEME* in combination with enzalutamide on PC-3 cell migration. Micrography and statistical analysis of the wound-healing assay. Values are expressed as mean ± S.E.M. Values with different letters (a–c) are significantly different (*P* < 0.05). Experiments were performed at least three times.

A wound-healing assay was performed for the PC-3 cells treated with a combination of *Stevia* extracts and enzalutamide. A total of 1000 μg/mL (the maximum non-cytotoxic concentration in fibroblasts) of the extracts with 40 μM of enzalutamide was used. *SPME* treatment alone showed a migration rate of 54%, 70%, and 100% at 24, 48, and 72 h, respectively. The combination of *SPME* with enzalutamide inhibited the closing of the scratch at all of the times evaluated, showing inhibition of migration rates of 40%, 42% and 45% at 24, 48, and 72 h, respectively. The combined treatment significantly increased the inhibition of closure of the wound compared to *SPME* alone (Figure 8). The treatment with *SEME* alone produced a migration rate of 54%, 77%, and 100% at 24, 48, and 72 h of exposure, respectively. The combination of *SEME* with enzalutamide inhibited the closing of the scratch at all of the times evaluated, and the migration rates were 42%, 54%, and 56% at 24, 48, and 72 h, respectively. The combined treatment of *SEME* with enzalutamide significantly increased the inhibition of the closure of the wound compared to *SEME* alone (Figure 9).

## 4. Discussion

The results showed that both *SPME* and *SEME* have no cytotoxic effect on human fibroblastic cells at concentrations ranging from 250 to 1000 μg/mL, and these results are consistent with the report on peripheral blood mononuclear cells exposed to *Stevia rebaudiana* extract [15]. In this project, the *Stevia* extracts were able to reduce the viability of the LNCaP and PC-3 cancer cells at all concentrations evaluated, and this effect is similar to that observed for *Stevia rebaudiana* extracts in breast cancer (MCF-7 and MDA), colon cancer (Caco2 and HCT116), lung cancer (A-549), cervical cancer (He-La), and pancreatic cancer cells (MiaPaCa-2) [10,16]. There are no previous reports on *Stevia pilosa* and *Stevia eupatoria* and their effects on cancer cells. However, their chemical composition has been reported, which helped us to explain their effects. *Stevia pilosa* and *Stevia eupatoria* extracts have compounds such as luteolin, quercetin, β-sitosterol, stigmasterol [11], *seco*-triterpenes [17], and longipinenes [18]. Luteolin has shown cellular arrest and apoptosis induction in many types of cancer cell lines, such as prostate cancer (PC-3), liver cancer (SMMC7721), colon cancer (COLO205), and cervical cancer (HeLa) [19]. Quercetin decreases the viability and proliferation of MCF-7 breast cancer cells through apoptosis activation, via increasing the levels of Bcl-2-associated X protein (BAX) and caspase-3 expression and decreasing Bcl-2 expression. In addition, quercetin activates necroptosis, via increasing the levels of receptor-interacting serine/threonine-protein kinase 1 (RIPK1) and receptor-interacting serine/threonine-protein kinase 3 (RIPK3) expression [20,21]. Both luteolin and quercetin were tested in prostate cancer cells DU145, showing cell cycle arrest [21]. Other compounds of *Stevia pilosa* and *Stevia eupatoria* include β-sitosterol and stigmasterol, which have an antiproliferative effect by activating the extracellular receptor kinase (ERK) 1/2 pathway, and apoptosis induction, decreasing Bcl-2 expression and increasing BAX expression in breast cancer cells MDA-MB-231 [22]. Additionally, some triterpenes demonstrated cytotoxicity in several cells such as colon and stomach cancer cells, inducing apoptosis via extrinsic and intrinsic pathways and activating caspase-8, -9, and -3 [23], and HEp-2, HCT 116, MCF-7, A-549, and PC-3 cancer cells [24]. Fraction compounds, mainly obtained by pinenes, showed a cytotoxic effect in A2058, MCF-7, HL-60, and HeLa cancer cells lines [25]. Some longipinene derivates displayed cytotoxicity on lung carcinoma H1299 cells [26]. No additive effect was observed with the combination of *Stevia* extracts and enzalutamide. This could be because enzalutamide has a mechanism of action both on the androgen receptor pathway and on caspase-8 and caspase-3 activation [22]. In LNCaP prostate cancer cells, enzalutamide shows overexpression in caspase-8 and caspase-3, such as luteolin, quercetin, stigmasterol, and β-sitosterol [27]. Therefore, we suggest that the effect on cell apoptosis did not increase with the combination because competition could have occurred between the compounds rather than an addition of effects by different mechanisms; however, more studies are needed to verify this hypothesis.

No studies of the effect of *Stevia* on the migration of cancer cells have been conducted; however, studies have examined the compounds found in this genus. Awad et al. found that β-sitosterol decreases invasion of PC-3 prostate cancer cells in vitro. A reduction of metastases to lymph nodes and to the lungs was observed, although the pathways through which phytosterol creates this effect were not analyzed [28]. Previous work showed that luteolin has an effect on metalloproteinases (MMPs), which are necessary for the degradation of the basement membrane, thus allowing invasion and migration to secondary sites. Luteolin in glioblastoma cell lines U251MG and U87MG decreases levels of MMP-2 and MMP-9 protein. In addition, luteolin has an effect on epithelial–mesenchymal transition (EMT), decreasing the protein expression of mesenchymal markers such as N-cadherin, Vimentin, and β-catenin, and increasing the protein expression of E-cadherin, which participates in cell–cell adhesion [29,30,31]. Quercetin is able to reduce the migration and invasion of human skin melanoma cells SK–MEL–28, increasing the expression of epithelial markers and decreasing mesenchymal markers [32]. The combination of *Stevia pilosa* and *Stevia eupatoria* with enzalutamide did not induce an antagonist effect on the migration of cells, which is consistent with wound-healing assay with enzalutamide alone reported by Khurana, et al. [33], and different from the antagonist effect reported by the same group with enzalutamide and sulforaphane in resistant cells [34]. Based on the above, we suggest that the extracts could affect some of these mechanisms, however, additional studies are needed.

## 5. Conclusions

The results indicated that *SPME* and *SEME* have an inhibitory effect on the viability and migration in prostate cancer cells, and although there is no evidence of the extracts increasing the anti-migratory effect of enzalutamide, the data suggest that *Stevia* extracts may represent a potential source of molecules for the treatment of cancer.
